# Colorectal Cancer Survival in 50- to 69-Year-Olds after Introducing the Faecal Immunochemical Test

**DOI:** 10.3390/cancers12092412

**Published:** 2020-08-25

**Authors:** María Angeles Gutierrez-Stampa, Vanessa Aguilar, Cristina Sarasqueta, Joaquín Cubiella, Isabel Portillo, Luis Bujanda

**Affiliations:** 1 Altza Primary Care Health Center, OSI Donostialdea; Biodonostia Health Research Institute, 20014 San Sebastián, Spain; MARIAANGELES.GUTIERREZSTAMPA@osakidetza.eus (M.A.G.-S.); VANESSA.AGUILARGAMA@osakidetza.eus (V.A.); 2 Biodonostia Health Research Institute, Red de Investigación en Servicios de Salud en Enfermedades Crónicas (REDISSEC), Hospital Universitario Donostia, 20014 San Sebastian, Spain; CRISTINA.SARASQUETAEIZAGUIRRE@osakidetza.eus; 3 Gastroenterology Department, Complexo Hospitalario Universitario de Ourense, 32005 Ourense, Spain; Joaquin.Cubiella.Fernandez@sergas.es; 4 Colorectal Cancer Screening Programme, Osakidetza, Basque Health Service, 48010 Bilbao, Spain; MARIAISABEL.PORTILLOVILLARES@osakidetza.eus; 5 Gastroenterology Department, Osakidetza, Hospital Universitario Donostia, Biodonostia Health Research Institute, Centro de Investigación Biomédica en Red en Enfermedades Hepáticas y Digestivas (CIBERehd), University of the Basque Country (UPV/EHU), 20014 San Sebastián, Spain

**Keywords:** colorectal cancer, faecal immunochemical test, survival

## Abstract

Population screening has improved early diagnosis of colorectal cancer (CRC). Nonetheless, most cases are diagnosed in symptomatic patients. Faecal immunochemical testing has been recommended for assessing patients with lower gastrointestinal symptoms, but whether it improves patient survival is unknown. Our objective was to compare CRC survival in 50- to 69-year-olds between asymptomatic screen-detected patients and symptomatic patients by route to diagnosis. Methods: We identified all cases of CRC diagnosed in 50-to 69-year-olds between 2009 and 2016, in Donostialdea (Gipuzkoa, Spain). Three groups were created: 1-screen-detected CRC; 2-CRC detected in symptomatic patients after a positive faecal immunochemical test(FIT); and 3-CRC detected in symptomatic patients without a FIT or after a negative result. We analysed survival using the Kaplan-Meier method and log-rank tests. Results: Of 930 patients diagnosed with CRC, 433 cases were detected through screening and 497 in symptomatic patients, 7.9% after a positive FIT and 45.5% by other means. The 3-year CRC survival was significantly lower in group 3 (69.5%) than groups 1 (93%; *p* = 0.007) or 2 (87.5%; *p* = 0.02). The risk of death was lower in groups 1 (HR 0.42, 95% CI 0.30–0.58) and 2 (HR 0.51; 95% CI 0.29–0.87). Conclusion: Half of CRC cases in 50- to 69-year-olds are diagnosed outside screening. Use of the FIT as a diagnostic strategy in symptomatic patients may improve survival.

## 1. Introduction

Colorectal cancer (CRC) is the third most common malignant cancer in Europe, when combining both sexes, and the second leading cause of cancer-related death in Spain [1]. Tumour stage at diagnosis is the best predictor of survival. For this reason, all efforts must be directed towards achieving a diagnosis in the early-stages, either through population-based screening programmes or prompt diagnosis in symptomatic patients.

The implementation of population screening programmes has helped achieve early diagnosis of CRC and reduced CRC-related mortality [2,3] by 18–57% depending on the type of test used [4]. In accordance with European recommendations and the strategy of the Spanish National Health System (2003) [5,6], the Basque Country implemented a population screening programme targeting 50- to 69-year-olds in 2009. Nonetheless, despite the implementation of such programmes, most CRC cases are still diagnosed in symptomatic patients [7,8]. Unfortunately, symptoms have low positive predictive value for CRC (3–4%), and hence, are not very useful for diagnosing this type of cancer. 

The gold standard for the detection of CRC is colonoscopy but this procedure is invasive, expensive and not complication free. In recent years, it has been shown that the faecal immunochemical test (FIT) is a tool that makes it possible to identify patients at the highest risk of CRC among those with symptoms [9,10]. FIT is a non-invasive test, which detects faecal haemoglobin concentrations (f-Hb), and has a high diagnostic accuracy for CRC [10,11,12], higher than that of the SIGN or NICE criteria [13] and traditional guaiac based faecal occult blood tests [14]. This test, with high sensitivity and specificity is essential for early detection and avoidance of unnecessary invasive tests. For this reason, the National Institute for Health and Care Excellence (NICE DG30) recommends its use in the assessment of patients with lower gastrointestinal symptoms [15,16] but who do not meet the criteria for a suspected cancer pathway referral. 

Nowadays, there are several studies that show a greater CRC survival in asymptomatic people by screening programmes but there is no data in literature about the impact of FIT in CRC survival in symptomatic patients. Recently our team has observed that FIT is still not widely used in primary care consultations in our region, however, those symptomatic patients diagnosed after a positive FIT had a higher survival [17]. 

The objective of this study was to assess the impact of the population screening programme on the diagnosis of CRC among people aged between 50 and 69 years old and compare CRC survival in this age range between screen-detected patients and symptomatic patients considering whether the FIT had been used in the diagnosis.

## 2. Results

Between 2009–16, 930 cases of CRC were recorded in people aged between 50 and 69 years old. Their mean age was 62 years and 61.8% of patients were male. The disease was detected in the distal colon or rectum in 71.3% and localized (stage I or II) in 60% of cases (Table 1).

### 2.1. Cases of CRC Diagnosed Through Population Screening (Group 1)

**Figure 1:**
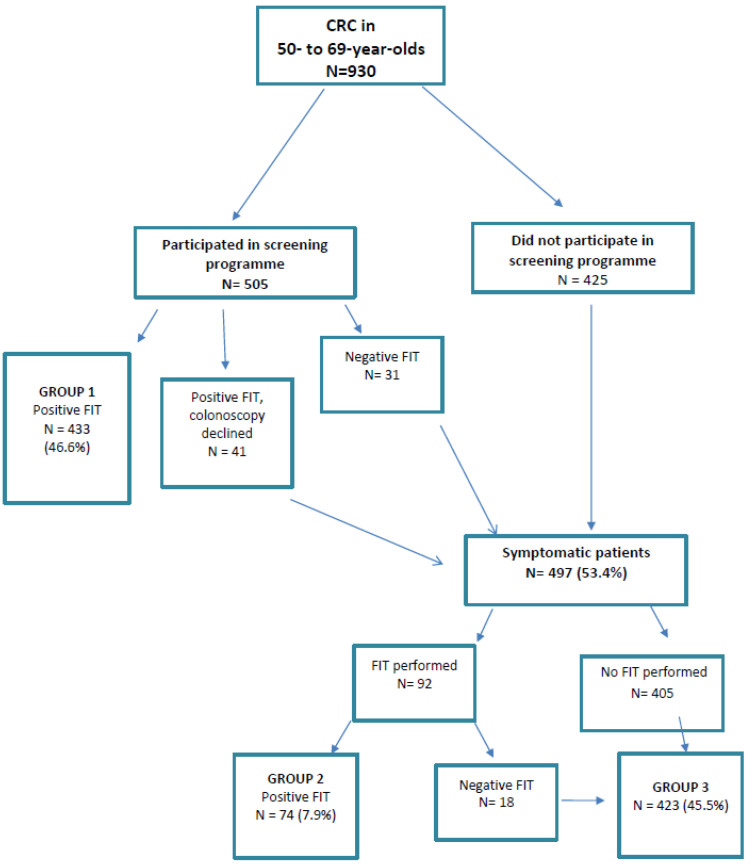
Flow diagram showing route to diagnosis of colorectal cancer (CRC) in groups 1, 2 and 3.

Out of the 930 cases of CRC, 505 were in patients who had participated in the screening programme (Figure 1).

Group 1: 433 cases of CRC (46.6%) diagnosed through the screening programme after a positive FIT result.

Additionally, there were: 41 cases (4.4%) in patients who had a positive FIT result but declined colonoscopy and were subsequently diagnosed when they presented with symptoms. 31 cases (3.3%) in patients who had a negative FIT result and were diagnosed less than 2 years later after having developed symptoms (i.e., cases of interval cancer in patients from the screening programme).

### 2.2. Cases of CRC Diagnosed in Symptomatic Patients (Groups 2 and 3)

Out of the 930 cases of CRC, 497 (53.4%) were diagnosed when patients already had symptoms. A total of 92 patients underwent a FIT following the appearance of symptoms (Figure 1).

Group 2: 74 cases of CRC (7.9%) diagnosed after a positive result in a FIT requested after symptom onset.

Group 3: 423 cases of CRC (45.5%), 405 diagnosed in patients who had not undergone a FIT (despite symptoms) and 18 in patients who had had a negative FIT result in the year before diagnosis.

### 2.3. Stage and Survival by Group

The clinical characteristics of patients are summarised in Table 2 by group. In the distribution of stages, we found significant differences between the three groups. Notably, 71.6% of the cases of screen-detected CRC were localized (stages I and II) at diagnosis compared to 52.7% and 49.2% of the cases of CRC in groups 2 and 3, respectively (*p* = 0.0005). However, although we may observe differences in stage distribution in both groups 2 and 3, there is no statistical significance. When considering only stage I, group 2 presents a 7% increase of stage I cases, when compared with group 3, although no statistical signifcance was observed (*p* = 0.17).

**Figure 2:**
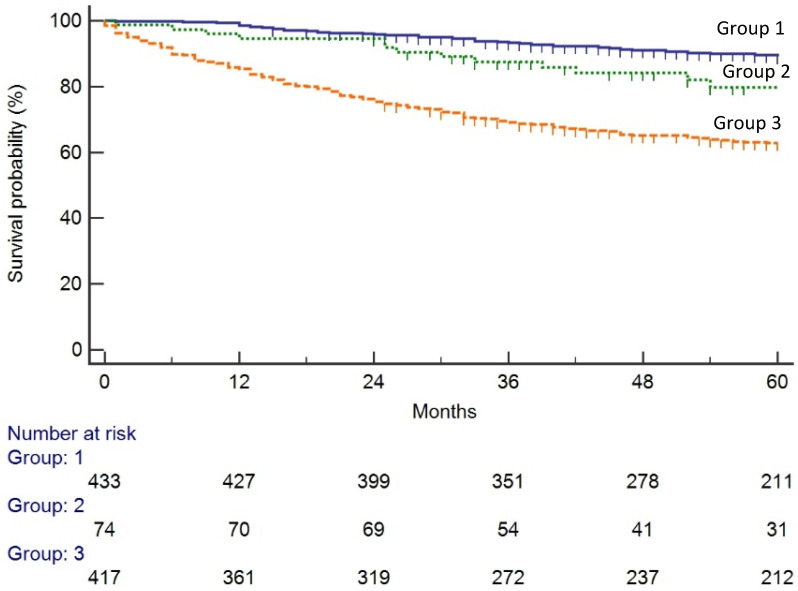
Kaplan-Meier overall survival curves by group. (Group 1: screen-detected CRC; Group 2: symptomatic patients with positive faecal immunochemical test results in the previous 12 months; Group 3: symptomatic patients who did not undergo faecal immunochemical testing or had a negative FIT result).

The 3-year survival was significantly lower in group 3 (69.5%) than group 2 (87.5%; *p* = 0.007) or group 1 (93%; *p* < 0.0005) and these differences remained significant at 5-year survival. (Table 2). (Figure 2) After adjusting for confounding variables (age and stage at diagnosis), the risk of death was lower in symptomatic patients diagnosed after a FIT (group 2, HR 0.51: 95% CI 0.29–0.87, *p* = 0.01); and in screen-detected patients (group 1, HR 0.42, 95% CI 0.30–0.58, *p* = 0.0005) (Table 3).

Table 3 lists the results of the multivariate analysis of the variables that influenced patient survival. Advanced stage, age and being in group 3 were found to be associated with poorer survival.

### 2.4. Cases of CRC in Patients with Positive FIT in Screening Who Declined Colonoscopy

We identified 41 patients with CRC who had had a positive FIT result in the screening but did not undergo colonoscopy after the FIT and were subsequently diagnosed after the onset of symptoms. The reasons for these colonoscopies not being performed were not medical; rather, patients declined the procedure. In this group, 58% of patients were male and the disease was detected in the proximal colon in 39%. Overall, 51.2% of these patients had advanced-stage CRC. Specifically, 77.8% (7/9) of the patients diagnosed with CRC more than 5 years after the FIT were in advanced stages compared to 44% (14/32) of those diagnosed within 5 years. The 3-year survival rate was 75.6%, significantly lower than the rate in the screen-detected group (93.5%) (*p* = 0.0005) (Table 4).

### 2.5. Interval Cancers

We detected 31 cases of interval cancer, this representing 6% of the 505 patients who had participated in the screening programme. In this group, 54.8% of patients were female, 38.7% had disease detected in the proximal colon and 55% had advanced-stage CRC. Notably, the rate of advanced-stage disease was 61% among the 18 cases diagnosed more than 12 months after their FIT but just 38% among those diagnosed within 12 months. The 3-year survival rate was 74.2%, significantly lower than the rate in the screen-detected group (93.5%) (*p* = 0.0005) (Table 4).

## 3. Discussion

### 3.1. Main Findings

This study analyses survival of CRC patients aged between 50–69 years by route to diagnosis and shows a higher survival rate in patients diagnosed after faecal immunochemical testing, both performed as part of the population screening programme and requested for individuals who seek medical attention for symptoms. Indeed, most cases of CRC in patients in this age range are still diagnosed outside the screening programme. On the other hand, patients with interval cancer and those who do not undergo the colonoscopy after a positive FIT have a higher mortality rate than patients diagnosed through the screening programme.

The target population of the Basque screening programme is 50- to 69-year-olds. The programme has a high rate of participation (69%), above the target recommended in European guidelines (65%), and the rate of colonoscopy acceptance is 92%. Despite this, almost half of CRC patients between 50–69 years old (45.6%) had never undergone a FIT for screening. In our analysis, we observed that only 46.6% of all 50- to 69-year-old patients with CRC are diagnosed through the population screening programme. Even in this age range, the focus of our screening programme, over half of patients are symptomatic at diagnosis. These data indicate that a high percentage of patients are missed by the screening programme, and moreover, that they are individuals who are at high risk of CRC. One of the reasons for this may be that the screening participation rate is lower in men, while the rate of malignant lesions is 3-fold higher in men than women, as it is already known [18,19,20]. Additionally, rates of participation in these programmes and of malignant lesions also vary with socioeconomic status [3,21,22,23,24]. For these reasons, to improve the performance of screening programmes, it is essential to increase participation rates, especially among men and individuals from lower socioeconomic groups, as has been observed in previous studies [25,26,27].

In this study, we found that the 5-year CRC survival differs by route to diagnosis (89%, 77% and 63% in groups 1, 2 and 3, respectively). Notably, survival is higher in patients diagnosed through the screening programme. It is already known that CRC population screening programmes have reduced CRC-related mortality [1,2,28], although most studies have been based on programmes using guaiac-based faecal occult blood tests. According to the systematic review of Gini et al [29], to date, only two observational studies from Italy have provided evidence that faecal immunochemical testing reduces mortality [30,31]. 

On the other hand, our data indicate that, in symptomatic patients, CRC survival is higher when the FIT is used for the diagnosis, nearly as high as that observed in patients diagnosed through the screening programme. It would be logical to think that that these lower mortality rates in the screen-detected group (Group 1) and symptomatic patients who had had a positive FIT (Group 2) are mainly attributable to the higher percentage of localized disease at the time of diagnosis in these groups. In fact, in our study, we found significant differences in the distribution of stages between the group 1 and the other study groups. Notably, the disease was localized (stage I or II) at diagnosis in 71.6% of screen-detected cases (Group 1), in line with previous studies [32,33,34], and in 52.7% of cases in symptomatic patients with a positive FIT (Group 2). Further, the rates of metastatic CRC were lower in these groups.

However, although a higher percentage of localized stages was observed in group 2 when compared with group 3, there is no statistical significance (Table 2). This result might be explained firstly by the size of our cohort. In this regard, we recently conducted a study including 1527 CRC patients with more than 18 years-old in which we were able to observe significant differences in stage distribution when comparing symptomatic patients that were diagnosed after a positive FIT result, with the other patients [17]. Secondly, there may be other factors, such as other comorbidities, that might affect the overall survival of the study group. Nonetheless, further studies with larger samples sizes are required to identify which patient- and medical-related factors have an impact on the decision of whether or not to request a FIT.

### 3.2. CCR in FIT-Positive Patients Who Declined Colonoscopy

Our study shows that 8.6% of patients diagnosed with CRC (*n* = 41) after a positive FIT result in the screening programme had not undergone the recommended colonoscopy. These results are consistent with those of the review by Domenech et al [35], who reported 7 to 12% of CRC patients failing to complete the diagnostic process, but lower than the rate found in the systematic review and meta-analysis of Gingold-Belfer et al [36], who observed that 19.4% of patients who obtained a positive FIT result in screening did not undergo a second diagnostic procedure. It is now clear that a colonoscopy should be performed after a positive FIT result to complete the screening process. Nonetheless, there are still relatively few studies that have analysed rates of adherence to recommendations in screening programmes and their impact. 

On the other hand, our data indicate that the 3-year survival rate is lower in these patients than those in the screen-detected group (75.6% vs. 93.5%). These data are in line with other studies [37] including that of Corley et al [38], based on a retrospective cohort in California, which observed a higher risk of advanced CRC stages in patients in whom a colonoscopy was performed more than 6 months after a positive FIT. In relation to this, according to the simulation model of Meester et al [39], a delay in diagnosis of more than 12 months after a positive FIT reduces the benefits of screening programmes. In our study, we found that patients who had declined a colonoscopy were diagnosed when they already had symptoms. Moreover, the rate of advanced-stage disease was higher among those diagnosed later. Given all this, it seems clear that we should underline the need to complete the diagnostic process in FIT-positive patients, as they are at risk of having CRC.

### 3.3. Interval Cancer

We detected 31 patients with interval cancer (6.1%) among the 505 cases of CRC in patients who underwent FIT as part of the screening programme, a lower rate than that reported in other studies but similar to figures previously reported for the Basque screening programme. Among these cases of interval cancer, there were more women than men and the disease was more likely to be in the proximal colon, as seen in other studies [8,40]. The rate of advanced-stage disease at diagnosis was higher among patients with interval cancer than among those diagnosed through the screening programme, especially in cases diagnosed more than 12 months after a FIT. This is probably due to the disease having developed over a longer period before detection. On the other hand, these patients also had a lower overall 3-year survival than patients in the screen-detected group. These results are in line with other studies [33,34] and may be related to a rapid growth and higher aggressiveness of these tumours.

### 3.4. Strengths and Weaknesses

This is one of the first studies to assess whether use of the FIT changes the prognosis of CRC in symptomatic patients aged between 50 and 69 years, compared to outcomes in other patients with CRC. Further, there are still relatively few studies that have focused on the impact of FITs for screening in terms of both diagnosis and mortality, in this age range. Another notable feature of the study is that it was carried out at population level and over a long period of time (7 years).

We should recognise, however, that our study has limitations. Given that it was retrospective, we were not able to obtain data on patient comorbidities or other risk factors such as personal or family history that may be important when comparing groups. 

We do not really know if the population of groups is really different or not. However, we have analysed that it is no differences in age, sex, histological variants and tumour site.

We do not know which factors associated with patients or doctors influence the decision of whether (or not) to request a FIT. It must take into account that FIT was implemented in our region in 2009 in Primary Care and at the beginning few doctors used it.

We were not able to accurately determine how Group 3 patients were diagnosed (in the emergency department, during hospital admission, or in an outpatient setting), the type of symptoms they experienced or the time from symptom onset to diagnosis. It is likely that some patients in this group had clear symptoms or increased comorbidity warranting direct referral for colonoscopy. Lastly, in some groups, the sample size was relatively small.

Therefore, although our results show that CRC survival was greater when diagnosed after a positive FIT result in symptomatic patients, we cannot explain the factors underlying the better prognosis. We have some limitations of making conclusions on causality because there may be biases in estimates due to residual confounding. So, we think further research with larger samples sizes are needed to confirm our results and, if they are confirmed, investigate the factors underlying the better prognosis. 

### 3.5. Implications for Research and Practice

Most cases of CRC are diagnosed when patients already have symptoms, even in the age range of 50–69 years, the target range for the screening programme. On the one hand, as screening allows identification of the disease before symptom onset and has shown to reduce CRC-related mortality, we should strengthen strategies for increasing patient participation, focusing on those at the highest risk of developing CRC. In fact, a high participation rate is essential to achieve the benefits of screening programmes. Moreover, we must underline the importance of performing colonoscopy examinations after a positive FIT result to achieve the best outcomes.

On the other hand, we must strengthen efforts to achieve an early diagnosis when CRC is diagnosed in individuals who already have symptoms. We should probably pay more attention not only to patients with warning signs but also to those with lower gastrointestinal symptoms (NICE) in whom a FIT may be useful to speed up the diagnosis. A great benefit of faecal immunochemical testing is that it achieves diagnosis at early stages, and for this reason, it seems advisable to encourage the use of FIT in primary care. Nonetheless, studies with larger samples are required to confirm these findings and, and if confirmed, determine which factors explain the better prognosis of CRC patients diagnosed after a FIT.

## 4. Materials and Methods 

### 4.1. Study Population

This was a retrospective cohort study. We identified all cases of CRC in 50- to 69-year-olds entered in the tumour registry of Donostia University Hospital (Gipuzkoa, Spain), between 2009 and 2016. We selected patients from the Donostialdea Health Region, with a catchment population of 360,000 and 30 healthcare centres.

CRC was diagnosed when neoplastic cells had passed through the muscularis mucosae, invading the submucosae (≥pT1). Patients were then excluded if they had CRC in situ, or cancers with histological features of a non-colon origin (melanoma, lymphoma). Stage 0 lesions, with high-grade dysplasia, intraepithelial neoplasia or intramucosal carcinoma were considered carcinoma in situ.

We classified patients into three groups as a function of the route by which CRC had been detected and analysed several different variables in each group. 

### 4.2. Design and Groups as a Function of Route to CRC Detection

We identified all FITs requested between 2009 and 2016 in our health region, all of these having been processed at Donostia Hospital laboratory, which is the referral laboratory for this region. The system used for testing for occult blood in our region is the OC-Sensor® (Eiken Chemical, Tokyo, Japan), an immunochemical test for the specific detection of human haemoglobin with a cut-off for positivity ≥10 μg Hb/g and using a single sample. The cut-off f-Hb was as recommended in NICE DG30 [16], results <10 μg Hb/g faeces being reported as “f-Hb not detected”. In our regional screening programme, a FIT result was considered positive if f-Hb was ≥20 μg Hb/g faeces. The results of this analysis are assessed qualitatively (positive or negative).

Additionally, we obtained data on population screening from all health centres in the Donostialdea Health Region between 2009 and 2016. The Donostialdea population screening programme was initiated in 2009 with a biennial FIT and colonoscopy for FIT-positive individuals, targeting all 50- to 69-year-olds. By 2014, the programme had reached 100% of the population. In 2015, 85% of the population had been called for screening at least twice and 56% three times and the mean participation rate was 69% [3].

Data from different databases were cross-checked, and among all cases of CRC, patients were classified into one of three groups as a function of the route to diagnosis:

Group 1: asymptomatic patients diagnosed through the CRC screening programme after a positive FIT result.

Group 2: symptomatic patients who had a positive FIT result within 12 months before the diagnosis.

Group 3: symptomatic patients who did not undergo faecal immunochemical testing or had a negative FIT result in the 12 months prior to their CRC diagnosis. 

Among all symptomatic patients, we stratified them into two groups based on whether they had performed a FIT in the year before the diagnosis of CRC. Then, we aimed to identify the patients who had been diagnosed after a positive FIT, and, subsequently, we aimed to compare the survival and other variables with the other study groups.

Further, we recorded all patients with CRC who had participated in the screening programme and had a negative FIT result in the 2 years prior to their diagnosis (interval CRC). We also identified all the cases of CRC in patients who participated in the screening programme and had a positive FIT result, but who did not undergo colonoscopy.

### 4.3. Variables

We analysed the following variables: age, sex, histopathological results, and CRC site, stage at diagnosis, and survival, as well as the results of FITs if performed. Patients were monitored until 31 December 2018. Histological variants were grouped into the following categories: adenocarcinoma, mucinous adenocarcinoma, and “others” (signet ring cell carcinoma, neuroendocrine carcinoma, or squamous cell carcinoma). The site of the CRC was classified as: proximal colon (caecum, ascending colon, hepatic flexure or transverse colon), distal colon (splenic flexure, descending colon and sigmoid colon) or rectum. The stage was defined in accordance with the TNM staging system [41]. Stages I and II were considered early stage and stages III and IV advanced. 

We analysed patient survival by documenting the date of diagnosis of CRC and the date of death if the patient died or confirmation that they were alive on 31 December 2018. We compared 3-year survival between the three groups.

The study was approved by the Ethics Committee of Gipuzkoa (protocol code: AGS-SOH-2017-01). The study protocol complies with the ethical principles of the 1975 Declaration of Helsinki as reflected in a priori approval by the institution’s human research committee. All the data collected in this project were processed anonymously in strict accordance with current data protection legislation (Law 41/2002 of 14 November; Law 15/1999 of 15 December).

### 4.4. Statistical Analysis

A descriptive analysis of the data was performed. Qualitative variables were expressed as absolute number and percentages. Differences between qualitative variables were assessed with the Chi-squared test and ANOVA for the age. Five-year survival was assessed using the Kaplan-Meier method and compared between groups with the log-rank test. A Cox regression model was used to adjust survival differences between groups for the following confounders: age, sex, site, histology and stage. The proportional hazard assumption was explored graphically for the group variable using a log–log plot and they showed proporcionality. Variables with a *p* < 0.2 in the univariate analysis were included in the multivariate model. The risk associated with each variable of interest was expressed as a hazard ratio (HR) and 95% confidence interval (95% CI). The statistical software IBM SPSS v23 was used and a p value less than or equal to 0.05 indicated statistical significance.

## 5. Conclusions

Our study shows that the majority of 50- to 69-year-olds with CRC are diagnosed once they have already symptoms, despite the fact that our regional screening programme targets this age range. Additionally, CRC survival is higher when diagnosed through screening or after a positive FIT in symptomatic patients. Although we believe that these are very interesting results, further research with larger samples size is required to confirm these data and explore the reasons for this better survival.

## Figures and Tables

**Table 1:** Descriptive data on cases of colorectal cancer studied.

Variables	930 Patients *n* (%)
**Mean age, years (SD)**	61.93 (SD 5.2)
**SEX**	
Man	575 (61.8)
Woman	355 (38.2)
SITE *	
Rectum	213 (22.9)
Distal colon	450 (48.4)
Proximal colon	255 (27.4)
Colon (not specified further)	12 (1.3)
**HISTOLOGY**	
Adenocarcinoma	893 (96.0)
Mucinous adenocarcinoma	25 (2.7)
Other **	12 (1.3)
**ROUTE TO DIAGNOSIS**	
Group 1	433 (46.5)
Group 2	74 (8.0)
Group 3	423 (45.5)
**STAGE**	
Stage I	309 (33.2)
Stage II	248 (26.7)
Stage III	210 (22.6)
Stage IV	162 (17.4)
Unknown	1 (0.1)

SD: standard deviation. * proximal colon: caecum, ascending colon, hepatic flexure or transverse colon; distal colon: splenic flexure, descending colon and sigmoid colon. ** Other: signet ring cell carcinoma, neuroendocrine carcinoma, squamous cell carcinoma. (Group 1: screen-detected CRC; Group 2: symptomatic patients with positive faecal immunochemical test results in the previous 12 months; Group 3: symptomatic patients who did not undergo faecal immunochemical testing or had a negative FIT result).

**Table 2:** Clinical characteristics of patients by group.

Variables	Group 1	Group 2	Group 3	*p*
	*n* = 433	*n* = 74	*n* = 423	
	*n* (%)	*n* (%)	*n* (%)	
AGE, years (SD)	61.8 (SD 5.3)	61.2 (SD 5.4)	62.1 (SD 5.1)	0.28
**SEX**				
(% MEN)	264 (60.9)	48 (64.8)	263 (62.2)	0.8
SITE *				
Rectum	79 (18.2)	21 (28.4)	113 (26.7)	
Distal	235 (54.3)	28 (37.8)	187 (44.2)	Group1/group2: 0.05
Proximal	116 (26.8)	24 (32.4)	115 (27.2)	Group2/group3: 0.71
Colon (not specified further)	3 (0.7)	1 (1.4)	8 (1.9)	Group1/group3: 0.003
**HISTOLOGY**				
Adenocarcinoma	423 (97.7)	71 (95.9)	399 (94.3)	Group1/group2: 0.03
Mucinous adenocarcinoma	9 (2.1)	1 (1.4)	15 (3.6)	Group2/group3: 0.6
Others **	1 (0.2)	2 (2.7)	9 (2.1)	Group1/group3: 0.01
**STAGE**				
Stage I	217 (50.1)	18 (24.3)	74 (17.5)	Group1/group2: < 0.0005
Stage II	93 (21.5)	21 (28.4)	134 (31.7)	Group2/group3: 0.46
Stage III	91 (21.0)	19 (25.7)	100 (23.7)	Group1/group3 < 0.0005
Stage IV	32 (7.4)	16 (21.6)	114 (26.9)	
Unknown	-	-	1 (0.2)	
3-year survival	93.0%	87.5%	69.5%	Group1/group2: 0.02 Group2/group3: 0.007 Group1/group3 < 0.0005
5-year survival	89%	77%	63%	Group1/group2: 0.02 Group2/group3: 0.007 Group1/group3 < 0.0005

SD: standard deviation. * proximal colon: caecum, ascending colon, hepatic flexure or transverse colon; distal colon: splenic flexure, descending colon and sigmoid colon. ** Other: signet ring cell carcinoma, neuroendocrine carcinoma, squamous cell carcinoma. (Group 1: screen-detected CRC; Group 2: symptomatic patients with positive faecal immunochemical test results in the previous 12 months; Group 3: symptomatic patients who did not undergo faecal immunochemical testing or had a negative FIT result).

**Table 3:** Association of variables with overall survival (univariate and multivariate analysis).

Variables	UNIVARIATE ANALYSIS			MULTIVARIATE ANALYSIS		
	HR *	95% CI **	*p*	HR *	95% CI **	*p*
**GROUPS**						
Group 3	1			1		
Group 1	0.26	0.19–0.36	0.0005	0.42	0.30–0.58	0.0005
Group 2	0.51	0.3–0.85	0.009	0.51	0.29–0.87	0.01
**AGE, years**	1.03	1.007–1.06	0.01	1.04	1.009–1.06	0.009
**SEX**						
Men	1					
Women	0.88	0.67–1.15	0.35			
**SITES**						
Rectum	1			1		
Distal	0.72	0.52–0.99	0.04	0.95	0.68–1.31	0.73
Proximal	0.92	0.65–1.3	0.65	1.22	0.86–1.73	0.27
**HISTOLOGY**						
Adenocarcinoma	1					
Mucinous adenocarcinoma	1.48	0.76–2.86	0.25			
Other ***	1.42	0.53–81	0.49			
**STAGE**						
Stage I	1			1		
Stage II	1.72	1.03–2.87	0.004	1.33	0.78–2.26	0.29
Stage III	3.43	2.14–5.51	0.0005	2.71	1.66–4.42	0.0005
Stage IV	19.07	12.35–19.45	0.0005	14.32	9.03–22.78	0.0005

* HR: hazard ratio. ** CI: confidence interval. *** Other: signet ring cell carcinoma, neuroendocrine carcinoma, squamous cell carcinoma. (Group 1: screen-detected CRC; Group 2: symptomatic patients with positive faecal immunochemical test results in the previous 12 months; Group 3: symptomatic patients who did not undergo faecal immunochemical testing or had a negative FIT result).

**Table 4:** Clinical characteristics of patients diagnosed with colorectal cancer.

Variables	Group 1	FIT-Positive Patients Who Declined Colonoscopy	Interval Cancer	*p*
	*n* = 433	*n* = 41	*n* = 31	
	*n* (%)	*n* (%)	*n* (%)	
**Age, years**	61.8 (SD 5.3)	62.5 (SD 4.8)	63.6 (SD 4.3)	0.1
**SEX**				
**(% MEN)**	264 (60.9)	24 (58.5)	14 (45.2)	0.22
**SITE**				
**Rectum**	79 (18.2)	14 (34.1)	9 (29.0)	
**Distal**	235 (54.3)	10 (24.4)	10 (32.3)	
**Proximal**	116 (26.8)	16 (39.1)	12 (38.7)	0.001
**Colon (not specified further)**	3 (0.7)	1 (2.4)	-	
**HISTOLOGY**				
**Adenocarcinoma**	423 (97.7)	39 (95.1)	26 (83.9)	
**Mucinous adenocarcinoma**	9 (2.1)	2 (4.9)	3 (9.7)	
Other **	1 (0.2)		2 (6.4)	0.0005
**STAGE**				
**Stage I**	217 (50.1)	10 (24.4)	6 (19.3)	
**Stage II**	93 (21.5)	10 (24.4)	8 (25.8)	
**Stage III**	91 (21.0)	11 (26.8)	8 (25.8)	
**Stage IV**	32 (7.4)	10 (24.4)	9 (29.1)	0.0005
**3-year survival**	93.5%	75.6%	74.2%	0.0005

(Group 1: screen-detected CRC; cases detected after having declined colonoscopy despite a positive faecal immunochemical test result and cases of interval cancer). ** Other: signet ring cell carcinoma, neuroendocrine carcinoma, squamous cell carcinoma.

## Abbreviations

CRC	colorectal cancer
FIT	faecal immunochemical test
f-Hb	faecal haemoglobin concentrations
HR	hazard ratio
OR	odds ratio
CI	confidence interval
